# Classification of non-acute bronchial asthma according to allergy and eosinophil characteristics: a retrospective study

**DOI:** 10.1186/s13223-021-00546-1

**Published:** 2021-05-03

**Authors:** Yi Jiang, Ruoli An, Li Cheng, Qianru Yue, Hanwei Zhang, Yali Zhang, Xiaomei Kong, Hongxia Ma, Fang Chen, Yufeng Guo

**Affiliations:** 1 Department of Respiratory and Critical Care Medicine, The First Hospital of Shanxi Medical University, Taiyuan, Shanxi China; 2 The First Clinical Medical College of Shanxi Medical University, Taiyuan, Shanxi China; 3 General Internal Medicine Department, People’s Hospital of Wenshui County, Shanxi Province, Wenshui, Shanxi China

**Keywords:** Asthma, Allergy, Eosinophils, Fractional exhaled nitric oxide, Sputum cytology

## Abstract

**Background:**

Investigating the endotypes of the different asthma phenotypes would help disease monitoring, prognosis determination, and improving asthma management standardization. This study aimed to classify asthma into four endotypes according to the allergic and eosinophilic characteristics and explore the phenotypes (clinical characteristics, pulmonary functions, and fractional expired nitric oxide (FeNO)) of each endotype.

**Methods:**

This retrospective study included non-acute asthma patients treated at the First Hospital of Shanxi Medical University (05/2016–01/2018). The patients were classified into the eosinophilic allergic, eosinophilic non-allergic, non-eosinophilic allergic, and non-eosinophilic non-allergic asthma endotypes. Serum sIgE, lung function, FeNO, and induced sputum cytology were tested and compared among groups.

**Results:**

Of the 171 included patients, 22 had eosinophilic allergic asthma, 17 had eosinophilic non-allergic asthma, 66 had non-eosinophilic allergic asthma, and 66 had non-eosinophilic non-allergic asthma. Lung function measurements (FEV1%, FEF25%, FEF50%, FEF75%, and FEF25–75%) showed that airway dysfunction was worse in eosinophilic non-allergic asthma than in the other three endotypes (all P < 0.001). In allergic asthma patients, eosinophilic asthma had worse airway dysfunction than non-eosinophilic asthma (all P < 0.05). Similar results were found in non-allergic asthma (all P < 0.01). The FeNO levels in eosinophilic allergic asthma were higher than in eosinophilic non-allergic and non-eosinophilic non-allergic asthma (both P = 0.001).

**Conclusions:**

FeNO can objectively reflect eosinophilic airway inflammation in asthma. Endotypic classification of asthma patients regarding the allergic and eosinophilic characteristics is conducive to the effective management of patients with asthma.

## Background

Asthma is a chronic inflammatory disorder of the airways characterized by bronchial hyperresponsiveness (BHR) and reversible airflow limitation [1, 2]. The incidence of self-reported asthma is 7–10% in adults [1, 3], and most of them had asthma during childhood [1, 3]. Asthma triggers include allergens, medications (particularly aspirin and nonsteroidal anti-inflammatory drugs), and environmental factors such as tobacco smoke and occupational exposure [1, 4]. Complications include secondary bacterial or viral lower respiratory infections, acute BHR, uncontrolled eosinophilic inflammation, complications associated with the chronic use of oral glucocorticosteroids, respiratory failure, and, rarely, death [1, 5]. About 5–10% of the patients do not respond well to standard treatment and have a poor prognosis [6]. There is also high mortality in patients who require intubation, have a history of severe disease, and have specific psychosocial factors [1].

The pathogenesis and clinical manifestations of asthma are very complex and heterogeneous [7], showing phenotypes and endotypes. Common phenotypes include allergic asthma, non-allergic asthma, delayed onset asthma, etc. Phenotypes are distinguished in order to better understand the severity of asthma, the duration of acute exacerbation and other characteristics. However, the same phenotype may have different pathophysiological mechanisms, and phenotypes can be reclassified according to the pathogenesis by using characteristic molecular biomarkers, which are called "endotypes". At present, the endotype of asthma includes Th2 correlation and non-Th2 correlation. Th2 type is a process of immune inflammation. The allergen stimulates the body to activate Th2 cells and release cytokines such as IL-4, IL-5 and IL-13, which act on airway epithelial cells, eosinophils, B lymphocytes and other inflammatory cells, showing the characteristics of allergy and eosinophilic associated inflammation and good response to ICS treatment. However, non-Th2 type may release cytokines such as IL-17 and IFN-γ by activation of Th1 cells, and activate neutrophils such as IL-6, G-CSF and GM-CSF directly through CXCL8 or indirectly from airway epithelial cells, leading to neutrophilic inflammation and AHRs, including neutrophilic asthma, obesity-related asthma and smoking-related asthma [8]. In addition, eosinophils, neutrophils and mast cells stimulate chronic inflammation, leading to basement membrane fibrosis, excessive mucus secretion, smooth muscle hypertrophy and angiogenesis, which damage the airway wall [9].

Endotype investigation is an effective method to explore the heterogeneity of asthma. The currently defined asthma endotypes include allergic asthma, non-allergic asthma, eosinophilic asthma, neutrophilic asthma, obesity-related asthma, and late-onset asthma [1]. Allergic asthma is one of the most important endotypes and is mainly driven by Th2 immune responses [9]. Fahy et al. [10] demonstrated eosinophilic inflammation in about 50% of asthma patients without allergic conditions, and eosinophilic inflammation was evident in both allergic and non-allergic endotypes. Investigating the characteristics of the different asthma endotypes and the resulting phenotypes would help disease monitoring, prognosis determination, and improving asthma management standardization [11]. FENO is generated by inducible nitric oxide synthase, which is induced by IL-13. It has been used for the assessment of eosinophilic airway inflammation in asthma [12]. FeNO is an important tool for the diagnosis of asthma and for monitoring the response to treatments [13]. Nevertheless, the FeNO levels are generally not increased in non-eosinophilic inflammation [12].

This study aims to classify asthma into four endotypes according to the allergic and eosinophilic characteristics and explore the phenotypes (clinical characteristics, pulmonary functions, and FeNO values) of each endotype. The results could help develop individualized treatment strategies.

## Methods

### Study design and patients

This retrospective study included non-acute asthma patients treated between May 2016 and January 2018 at the Outpatient Department of Respiration of the First Hospital of Shanxi Medical University. This study was approved by the Ethics Committee of the First Hospital of Shanxi Medical University (#2019 K-K010). The requirement for informed consent was waived by the Committee.

The inclusion criteria were (1) met the diagnostic criteria of asthma [14]: history of variable respiratory symptoms; Confirmed variable expiratory airflow limitation. Positive bronchodilator (BD) reversibility Test (increase in FEV 1 of > 12% and > 200 mL from baseline, Positive bronchial challenge test (Fall in FEV 1 from baseline of ≥ 20% with standard doses of methacholine) (Table 1). (2) in a non-acute phase. Patients with one or more of the following characteristics were excluded: (1) pneumonia, pulmonary tuberculosis, bronchiectasis, or other respiratory diseases, (2) diseases that could increase the eosinophils or induce evident abnormalities of blood routine examination results, such as parasitic infection, allergic bronchopulmonary aspergillosis (ABPA), lymphoma, or leukemia, (3) other chronic diseases or conditions such as heart disease, hypertension, diabetes, pregnancy, or malignant tumors, or (4) currently on oral glucocorticoids treatment or anti-IgE drug treatment.

**Table 1:** Criteria for making the diagnosis of asthma

1. History of variable respiratory symptoms
Generally more than one type of respiratory symptom (in adults, isolated cough is seldom due to asthma) Symptoms occur variably over time and vary in intensity Symptoms are often worse at night or on waking Symptoms are often triggered by exercise, laughter, allergens, cold air Symptoms often appear or worsen with viral infections
2. Confirmed variable expiratory airflow limitation
At least once during diagnostic process (e.g. when FEV 1 is low), confirm that FEV 1 /FVC is reduced (normally > 0.75–0.80) Positive bronchodilator (BD) reversibility Test: increase in FEV 1 of > 12% and > 200 mL from baseline, 10–15 min after 200–400 mcg albuterol or equivalent (greater confidence if increase is > 15% and > 400 mL); Excessive variability in twice-daily PEF over 2 weeks: average daily diurnal PEF variability > 10%; Significant increase in lung function after 4 weeks of anti-inflammatory treatment: increase in FEV 1 by > 12% and > 200 mL (or PEF † by > 20%) from baseline after 4 weeks of treatment, outside respiratory infections; Positive exercise challenge test: fall in FEV 1 of > 10% and > 200 mL from baseline; Positive bronchial challenge test: Fall in FEV 1 from baseline of ≥ 20% with standard doses of methacholine, or ≥ 15% with standardized hyperventilation, hypertonic saline or mannitol challenge; Excessive variation in lung function between visits: variation in FEV 1 of > 12% and > 200 mL between visits, outside of respiratory infections

### Grouping

According to the allergic and eosinophilic characteristics, the asthma patients were classified into four endotypes: eosinophilic allergic asthma, eosinophilic non-allergic asthma, non-eosinophilic allergic asthma, and non-eosinophilic non-allergic asthma. The diagnostic criteria of allergic asthma were those of the GINA guidelines [1] and the Guidelines for Diagnosis and Treatment of Bronchial Asthma [14]: exposure to the allergen induces or aggravates the symptoms, and the skin prick test and serum specific IgE test show positive responses to at least one allergen. The diagnostic criteria of eosinophilic asthma were a cytological examination of sputum showing eosinophil percentage (EOS%) ≥ 3% and neutrophil percentage (NEU%) < 61% [15]. Hence, eosinophilic allergic asthma was diagnosed in the presence of specific IgE positive tests and cytological examination of induced sputum showing NEU% < 61% and EOS% ≥ 3% [16].

### Data collection

The clinical data of the treatment-naïve patients who met the inclusion criteria, including age, sex, body mass index (BMI), smoking history, clinical type, average diagnosis time, family history, complications, serum IgE levels, pulmonary function levels, FeNO levels, and cytological classification of induced sputum, were collected.

The cytological classification of induced sputum was performed according to the Standards of Inducing Sputum [17] issued by the Respiratory Society of the Chinese Medical Association in 2015. Ultrasonic atomization of 3% hypertonic saline was used to induce expectoration. The sputum was collected and processed, and 400 non-squamous epithelial cells were collected and submitted to cytological classification.

The FeNO measurement was performed according to the Guidelines for FeNO measurement [9] issued by the American Thoracic Society (ATS) and the European Respiratory Society (ERS). A Sunvou expiration analyzer (Sunvou-CA2122, Shangwo Medical Electronics Co., Ltd., Wuxi, Jiangsu, China) was used to measure the FeNO levels. The patients were asked to exhale as much as possible in the resting state, to wrap their lips tightly around the filter, to inhale calmly to total lung capacity, and then to exhale slowly. The exhalation airflow speed was maintained at 50 ml/s, and the exhalation plateau was kept > 2 s.

The pulmonary ventilation function was measured using a pulmonary function instrument (Jaeger AG, Wurzburg, Germany). The measurement was repeated three times, and the best results were recorded. The forced expiratory volume in one second as a percentage of the expected value (FEV1%) and the forced expiratory flow at 25%, 50%, and 75% vital capacity (FEF25%, FEF50%, and FEF75%) were measured.

The measurement of specific IgE was performed using the AllergyScreen system (Mediwiss Analytic GmbH, Moers, Germany) and the allergen sIgE kit (immunoblotting method), which provided a semi-quantification of allergen sIgE in the human serum. All procedures were performed strictly according to the manufacturer’s instructions. Allergen sIgE grade ≥ 1 (0.35 U/ml) was considered positive.

### Airway dysfunction measurement

FEV1% was used to assess airway dysfunction. The FEV1 tends to decrease when airway obstruction occurs [18]. The FEF25%, FEF50%, FEF75%, and FEF25%–75% were also measured. Airflow speed decreased at the end of forced expiration, and at least two among FEF75%, FEF50%, and FEF25%–75%, being < 65% of the expected values, suggested small airway dysfunction.

## Statistical analysis

SPSS 22.0 (IBM, Armonk, NY, USA) was used for statistical analysis. Continuous data with a normal distribution (according to the Levene test) are described as means ± standard deviations and were analyzed using a one-way analysis of variance (ANOVA) with the LSD post hoc test. Continuous data with a non-normal distribution are presented as medians (interquartile ranges (IQRs)) and were analyzed using non-parametric tests. Continuous data are described as n (%) and were compared with the chi-square test. P < 0.05 was considered statistically significant.

## Results

### Characteristics of the patients

Between May 2016 to January 2018, 171 patients with non-acute asthma were treated at the Outpatient Department of Respiration of the First Hospital of Shanxi Medical University. Among them, 22 (8 males and 14 females) had eosinophilic allergic asthma, 17 patients (4 males and 13 females) had eosinophilic non-allergic asthma, 66 patients (19 males and 57 females) had non-eosinophilic non-allergic asthma, and 66 patients (19 males and 47 females) had non-eosinophilic allergic asthma. Three (13.6%), four (23.5%), seven (10.6%), and 11 (16.7%) patients with eosinophilic allergic, eosinophilic non-allergic, non-eosinophilic non-allergic, and non-eosinophilic allergic asthma were smokers, respectively. The patients with non-eosinophilic non-allergic asthma (45.5 ± 12.9 years) were older than the patients with eosinophilic allergic asthma (33.9 ± 13.5 years; P < 0.001) and non-eosinophilic allergic asthma (38.6 ± 12.8 years; P = 0.003). BMI was similar among the four groups (P = 0.086) (Table 2).

**Table 2:** Demographic, clinical, and functional characteristics of the patients

Clinical features	Eosinophilic allergic asthma, n = 22	Eosinophilic non-allergic asthma, n = 17	Non-eosinophilic allergic asthma, n = 66	Non-eosinophilic non-allergic asthma, n = 66	P
Sex, n (M/F)	8/14	4/13	19/47	19/47	0.845
Age (years), means ± SD	33.9 ± 13.5*	39.2 ± 14.1	38.6 ± 12.8*	45.5 ± 12.9	0.001
Body mass index (kg/m^2^), means ± SD	25.3 ± 4.7	23.3 ± 3.7	24.1 ± 3.6	25.5 ± 4.3	0.086
Manifestation, n (%)					
CA	19 (86.4%)	15 (88.2%)	41 (62.1%)	30 (45.5%)	< 0.001
CVA	2 (9.0%)	2 (11.8%)	25 (37.9%)	35 (53.0%)	
CTVA	1 (4.6%)	0	1 (1.5%)	0	> 0.99
Exercise-induced asthma, n (%)	0	0	1(1.5%)	1(1.5%)	> 0.99
Average time to diagnosis (months), median	36.3	25.5	32.9	44.5	0.075
Comorbidity and history, n (%)					
Rhinitis/nasosinusitis	7 (31.8%)	6 (35.3%)	13 (19.7%)	8 (12.1%)	0.072
Other allergic diseases^a^	7 (31.8%)	2 (11.8%)	25 (25.8%)	17 (25.8%)	0.154
Other diseases^b^	4 (18.2%)	4 (23.5%)	35 (53.0%)	22 (33.3%)	0.007
Obesity	7 (31.8%)	1 (4.5%)	8 (12.1%)	19 (28.8%)	0.022
Smoking history, n (%)	3 (13.6%)	4 (23.5%)	7 (10.6%)	11 (16.7%)	0.486^e^
Family history of allergic diseases	2 (9.1%)	3 (17.6%)	13 (19.7%)	5 (7.6%)	0.188
Laboratory tests					
Eosinophil count induced sputum (%), median (IQR)	23.65 (14.65–39.88)	59.47 (23.40–86.40)	0.34 (0–1.53)^cd^	0.28 (0–1.50)^cd^	< 0.001
Neutrophil count induced sputum (%), median (IQR)	43.62 (30.70–53.15)	35.64 (10.45–42.52)	76.5^ cd^ (57.73–89.96)	79.54 (59.36–93.81)^cd^	< 0.001
Neutrophil count induced sputum (%), median (IQR)	43.62 (30.70–53.15)	35.64 (10.45–42.52)	76.5^ cd^ (57.73–89.96)	79.54 (59.36–93.81)^cd^	< 0.001
Total IgE serum, IU/mL, means ± SD	237.50 ± 13.76	130.24 ± 20.58	100.25 ± 10.43	80.65 ± 20.54	0.188
Eosinophil count peripheral blood (10^9^/L), median, IQR	0.35 (0.23–064)	0.49 (0.28–1.54)	0.11 (0.06–0.24)^cd^	0.11 (0.07–0.23)^cd^	< 0.001
Eosinophils in peripheral blood (%), median (IQR)	5.8 (3.3–8.8)	7.8 (5.85–14.33)	1.7 (0.98–3.65)^cd^	1.75 (1.05–2.85)^cd^	< 0.001

CA; classical asthma; CVA: cough variant asthma; CTVA: chest tightness variant asthma; IQR: interquartile range

^a^Including urticaria, eczema, allergic dermatitis, drug allergy, and food allergy

^b^Including anxiety, depression, and sleep apnea–hypopnea syndrome

^c^P < 0.001 vs. patients with eosinophilic allergic asthma

^d^P < 0.001 vs. patients with eosinophilic non-allergic asthma

^*^P < 0.05 vs. patients with non-eosinophilic non-allergic asthma

^e^Fisher’s exact test

### Clinical characteristics among the four endotypes of asthma

Among the patients with eosinophilic allergic asthma, 19 (86.4%), two (9.0%), and one (4.5%) had classical asthma (CA), cough variant asthma (CVA), and chest tightness variant asthma (CTVA), respectively. Among the patients with eosinophilic non-allergic asthma, 15 (88.2%) and two (11.8%) had CA and CVA, respectively. Among the patients with non-eosinophilic allergic asthma, 41 (62.1%), 25 (37.9%), and one had CA, CVA, and CTVA, respectively. Among the patients with non-eosinophilic non-allergic asthma, 30 (45.5%), 35 (53%), and one had CA, CVA, and CTVA, respectively. The percentage of CVA was significantly higher in the patients with eosinophilic non-allergic asthma, and CA was significantly higher in the patients with non-eosinophilic non-allergic asthma (P < 0.001) (Table 2).

### Comorbidities and family history among the four endotypes

Among the patients with eosinophilic allergic asthma, seven (31.8%) had rhinitis/nasosinusitis, seven (31.8%) had other allergic diseases, seven (31.8%) had obesity and four (18.2%) had other diseases. Among the patients with eosinophilic non-allergic asthma, six (35.3%) had rhinitis/nasosinusitis, two (31.8%) had other allergic diseases, one (4.5%) had obesity, and four (23.5%) had other diseases. patients with non-eosinophilic allergic asthma, 13 (19.7%) had rhinitis/nasosinusitis, 25 (25.8%) had other allergic diseases, 8 (12.1%) had obesity, and 35 (53.0%) had other diseases. Among the patients with non-eosinophilic non-allergic asthma, 8 (12.1%) had rhinitis/nasosinusitis, 17 (25.8%) had other allergic diseases, 19 (28.8%) had obesity, and 22 (33.3%) had other diseases. There were no differences in the proportion of patients with a family history of allergic diseases (P = 0.188) (Table 2).

### FeNO and pulmonary functions among the four endotypes

**Fig. 1:**
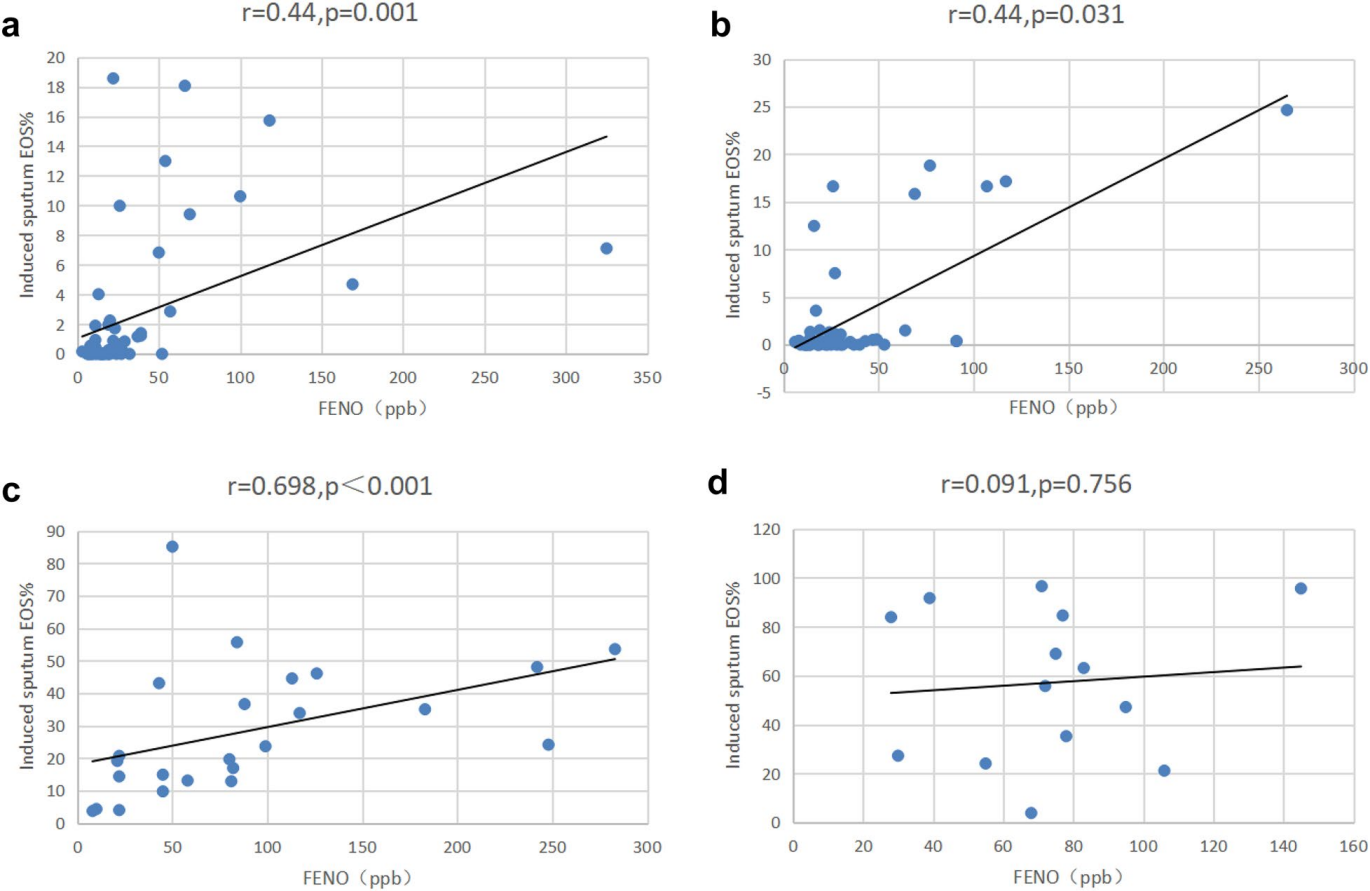
Association between sputum eosinophils (EOS) and exhaled nitric oxide (FeNO) in patients among the four endotypes. **a** Association between sputum EOS and FeNO in patients with non-eosinophilic allergic asthma. **b** Association between sputum EOS and FeNO in patients with non-eosinophilic non-allergic asthma. **c** Association between sputum EOS and FeNO in patients with eosinophilic allergic asthma. **d** Association between sputum EOS and FeNO in patients with eosinophilic non-allergic asthma

The FeNO values were significantly different among the four endotypes (P < 0.001). The FeNO values in patients with eosinophilic (irrespective of allergic) asthma were significantly higher than those with non-eosinophilic asthma. Pairwise comparisons showed that the FeNO values in patients with eosinophilic allergic asthma were higher than in eosinophilic non-allergic asthma and non-eosinophilic non-allergic asthma (P = 0.001 and P = 0.001, respectively) (Table 3). The pulmonary function indicators, including FEV1%, FEF25%, FEF50%, FEF75%, and FEF25–75%, were significantly poorer in patients with eosinophilic non-allergic asthma than in the other three endotypes (all P < 0.05). Among the patients with allergic asthma, the FEV1%, FEF25%, FEF50%, FEF75%, and FEF25–75% were all significantly poorer in those with eosinophilic allergic asthma compared with non-eosinophilic allergic asthma (P = 0.009, P = 0.001, P = 0.008, P = 0.004, and P = 0.033, respectively). Among the patients with non-allergic asthma, the FEV1%, FEF25%, FEF50%, FEF75%, and FEF25–75% were all significantly poorer in those with eosinophilic non-allergic asthma compared with non-eosinophilic non-allergic asthma (P = 0.001, P < 0.001, P < 0.001, P = 0.002, and P < 0.001, respectively) (Table 3). Figure 1 shows the correlations between FeNO and EOS% in the four groups.

**Table 3:** Comparison of pulmonary functions and FeNO levels among the four endotypes

Indicators	Eosinophilic allergic asthma, n = 22	Eosinophilic non-allergic asthma, n = 17	Non-eosinophilic allergic asthma, n = 66	Non-eosinophilic non-allergic asthma, n = 66	P
FEV1%					
(χ ± s)	97.15 ± 25.45^a^	81.55 ± 28.25	111.09 ± 20.92^ab^	104.66 ± 18.75^a^	< 0.001
FEF25%					
(χ ± s)	80.86 ± 39.74^a^	67.22 ± 38.30	108.82 ± 30.95^ab^	101.97 ± 30.01^a^	< 0.001
FEF50%					
(χ ± s)	74.71 ± 44.13^a^	52.42 ± 30.97	99.67 ± 38.98^ab^	94.84 ± 35.28^a^	< 0.001
FEF75%					
(χ ± s)	70.10 ± 42.51^a^	44.82 ± 27.90	79.52 ± 36.89^ab^	74.75 ± 31.78^a^	0.004
FEF25-75%					
(χ ± s)	69.83 ± 39.61^a^	48.50 ± 29.13	87.45 ± 34.68^ab^	83.53 ± 30.47^a^	< 0.001
Large airway dysfunction					
n (%)	10 (45.5)	15 (88.2)	10 (15.2)	18 (27.3)	< 0.001
Small airway dysfunction					
n (%)	12 (54.5)	10 (58.8)	30 (45.5)	28 (42.4)	0.5574
FeNO					
Median (IQR)	58 (21.5–112.5)	76 (64.75–97.75)	20 (13.5–37.5)^ab^	22.5 (13.75–37.75)^ab^	< 0.001

FEV1%: forced expiratory volume in one second as a percentage of expected value; FEF25%: forced expiratory flow at 25% vital capacity; FEF50%: forced expiratory flow at 50% vital capacity; FEF75%: forced expiratory flow at 75% vital capacity; FEF25–75%: forced expiratory flow at 25–75% vital capacity; FeNO: fractional exhaled nitric oxide; 1 ppb = l × 10^–9^; FEV: forced expiratory volume

^a^P < 0.05 vs. patients with eosinophilic non-allergic asthma

^b^P < 0.05 vs. patients with eosinophilic allergic asthma

## Discussion

Investigating the characteristics of the different asthma endotypes is an important step in improving disease monitoring and prognosis determination and knowing about individual asthma endotypes and planning personalized asthma management [11]. The phenotypes of asthma refer to the observed clinical characteristics, which are the results of the endotypes that involve environmental, clinical, and genetic factors [1, 19]. Bronchial asthma has various phenotypes, with allergic asthma being the most important one. It is induced by the IgE-mediated adaptive immune responses and non-IgE-mediated innate immune responses, of which eosinophils are the effector cell [20]. IgE and non-IgE cause eosinophils to enter the lungs through different pathogenesis and participate in immune inflammatory response [20, 21]. The endotypes of airway inflammation in asthma include eosinophilic asthma, neutrophilic asthma, and others [22, 23]. Li et al. [23] found that the airway inflammation endotype in asthma patients was mainly of the eosinophilic type and that serum IgE levels increased in some patients. These findings demonstrated that the phenotypes of different asthma endotypes could overlap. Therefore, this study aims to classify asthma into four endotypes according to the allergic and eosinophilic characteristics and explore the phenotypes (clinical characteristics, pulmonary functions, and FeNO) of each endotype.

The findings showed that the patients were significantly younger in the eosinophilic allergic asthma endotype, the age of onset is usually in childhood and the patients tended to be allergic to multiple allergens, and they also had a higher proportion of allergic comorbidities, including allergic rhinitis, sinusitis, eczema, urticaria etc. Terl et al. [24] developed joint national guidelines for managing asthma, according to the endotypes of asthma, which highlighted that the major characteristics of eosinophilic allergic asthma include evident allergic responses, disease onset mainly in childhood, and eczema and other allergic comorbidities or diseases, while severe asthma is generally accompanied by a fungal allergy. The studies have shown that allergic eosinophilic asthma is mainly characterized by allergy, and allergen removal therapy, allergen immunotherapy and targeted therapy (omalizumab) are adopted.

The findings of this present study also showed that FeNO can objectively reflect eosinophilic airway inflammation in asthma. The FeNO values in the patients with eosinophilic asthma, regardless of allergy status, were higher than in non-eosinophilic asthma and were the highest in the patients with eosinophilic allergic asthma. These findings suggested that as a new, non-invasive airway inflammation marker, FeNO can reliably reflect the eosinophilic airway inflammation, as supported by the literature [12, 13, 25, 26]. Nevertheless, Crespo et al. [27] reported that the FeNO levels in patients with eosinophilic non-allergic asthma (FeNO generally < 50 ppb) were significantly lower than in patients with non-eosinophilic allergic asthma (FeNO generally ≥ 50 ppb), which contradict our findings. Both FeNO and eosinophils are parts of the Th2 inflammation responses, but they are regulated by different inflammation pathways. FeNO is a reliable predictive factor reflecting Th2 inflammation and responses to glucocorticoids’ inhalation and could increase in patients with the phenotype of eosinophilic airway inflammation [25]. The activation of the Th2 inflammation cascade responses leads to the release of various cytokines, including IL-4 and IL-13, to regulate the synthesis of IgE and NO, while IL-5 could increase eosinophils [26]. Continuously increased eosinophils in induced sputum, as well as increased FeNO levels, both indicate an increased risk of acute asthma attacks [1]. Nevertheless, the association between eosinophils and FeNO needs to be further investigated.

Pulmonary function is an important indicator for assessing the symptoms of asthma patients, of which FEV1% is an important component in predicting future risk [1]. The present study showed that the functions of large and small airways were both poorer in patients with eosinophilic non-allergic asthma, compared with the other three endotypes. Sakagami et al. [28] used hierarchical cluster analysis to investigate asthma phenotypes with accelerated pulmonary function decrease. The patients’ mean disease duration was 17 years, and the patients were followed for 4 years. All patients received medications in accordance with the GINA guidelines. Finally, the patients were classified into three groups, of which the allergic characteristics were more prominent in Group 1 than in Groups 2 and 3, while the reduction of FEV1% was more pronounced in Group 3 than in the other two groups. These findings demonstrated that compared with allergic asthma, reducing the pulmonary function was more substantial in non-allergic asthma patients [28]. Although the present study also demonstrated that the pulmonary functions of patients with non-allergic asthma were relatively poorer than in the other endotypes, no longitudinal investigation was performed. In a study of patients with severe asthma, Moore et al. [29] performed a cluster analysis of patients older than 12 years and receiving inhaled corticosteroids and found that three clusters were allergic and eosinophilia were elevated, while one cluster was with decreased pulmonary function and required oral glucocorticoids. The results of this study are inconsistent with the conclusion, and the difference may be related to the disease severity and ethnicites of the included population.

Nevertheless, the allergy severity is not in agreement with pulmonary function but could be associated with the severity of asthma, ICS doses, and disease management. So it is important to classify and manage asthma according to induced sputum cytology. The findings of this study suggest that patients with eosinophilic asthma have poorer pulmonary functions than non-eosinophilic asthma patients, regardless of allergy conditions. Previous studies found that the pulmonary functions were worse in adult patients with critical asthma and higher eosinophil percentage in sputum [30, 31], suggesting that eosinophilic airway inflammation is associated with poorer pulmonary functions in asthma patients. An early open-label study showed that sputum eosinophil-based disease management could prevent the acute attack of asthma [32]. Therefore, eosinophils can be used to monitor and predict the activity and progression of asthma and the assessment of glucocorticoid treatment efficacy. The risk of an acute attack is relatively high for eosinophilic allergic asthma, which suggests that more attention on eosinophilic asthma is needed in clinical practice and that the eosinophil levels need to be regularly monitored during the treatment and management of asthma. In addition, induced sputum and FeNO levels could also be used to predict the severity of airway eosinophilic inflammation, according to which the treatment strategy could be adjusted to prevent the acute attack of asthma of the patients with this endotype.

Obesity-related asthma is a phenotype of asthma in which patients have significant respiratory symptoms but not significant eosinophilic airway inflammation. This study also suggests that many patients with non-eosinophilic non-allergic asthma were obese. Patients with non-eosinophilic non-allergic asthma did not have increased eosinophils or allergic symptoms, and the patients were generally older at disease onset. The severity of asthma is generally associated with obesity and other complications [19]. Frey et al. [33] demonstrated that the BMI of patients with asthma was higher than in individuals without asthma. More importantly, the respiratory symptoms of some obese patients with asthma are more evident, while the eosinophilic airway inflammation is mild [33]. The present study is in agreement with Frey’s findings. So we suggest that non-allergic and non-eosinophilic asthma may be obesity-related asthma, with significant respiratory symptoms and reduced eosinophilic airway inflammation. As a risk factor of asthma, obesity could induce or aggravate asthma. Thus, reducing body weight is one of the strategies for the management of asthma.

The purpose of further classification integrating allergy with eosinophil is to help assess endotypes and severity of asthma, which is the basis of appropriate treatment to achieve complete control. For patients with allergic eosinophilic asthma, the main characteristic is allergy. Patients with mild allergies may be given allergen immunotherapy, such as sublingual or subcutaneous desensitization. And for patients with severe allergic eosinophilic asthma the physician may prescribe targeted therapy in future, including anti-IgE treatment, anti-interleukin-5/5R treatment, anti-interleukin-4Rα treatment. For patients with non-allergic eosinophilic asthma, who have obvious characteristics of eosinophilic granulocyte increase, anti-interleukin-5/5R treatment or oral corticosteroids may be given in future when asthma is just partly controlled or even uncontrolled. The classification also suggests that allergic bronchopulmonary aspergillosis (ABPA) and eosinophilic granulomatosis polyangiitis (EGPA) may be considered in asthmatic patients with abnormally elevated airway inflammation of eosinophilic, and that should be noted during treatment. For non-eosinophilic and non-allergic asthma patients, who are often associated with obesity, obesity can lead to complications including diabetes, sleep apnea syndrome and cardiovascular disease etc., so it is important to lose weight and treat complications of non-eosinophilic and non-allergic asthma. A comprehensive asthma treatment strategy should be based on endotypes classification, with continuous assessment of induced sputum endotypes before initial treatment, after treatment, and during daily follow-up, as well as assessment of Allergen transition (allergens are reviewed every few years), so that the treatment strategy can be used to continuously optimize treatment.

This study has limitations. The number of samples was small and they were from a single center. Due to the retrospective nature of the study, the data that could be analyzed were limited to those available in the patient charts. Future studies should examine complete panels of inflammation and immunity markers.

## Conclusions

Achieving and maintaining the optimal control of asthma is an important target of asthma management. Endotype-oriented studies have an important significance in clarifying the pathogenesis, guiding the treatment, and predicting asthma prognosis [34]. Allergic and eosinophilic are two characteristics of Th2 inflammation. The present study described the disease characteristics of patients with four different endotypes based on these two characteristics, but the exact cellular and biological mechanisms remain to be investigated. The endotypes and phenotypes of asthma are very complex and are still unclear. Simpler and more applicable classification methods are needed in future clinical practices to help explore individualized treatments for patients with different asthma endotypes.

## Data Availability

All data generated or analyzed during this study are included in this published article.

## Abbreviations

BHR: Bronchial hyperresponsiveness
Th1: T helper 1
Th2: T helper 2
FeNO: Fractional exhaled nitric oxide
ABPA: Allergic bronchopulmonary aspergillosis
EOS%: Eosinophil percentage
NEU%: Neutrophil percentage
ANOVA: Analysis of variance
IQRs: Interquartile ranges
CA: Classical asthma
CVA: Cough variant asthma
CTVA: Chest tightness variant asthma
